# Silver Nanoparticles Supported onto TEMPO-Oxidized Cellulose Nanofibers for Promoting Cd^2+^ Cation Adsorption

**DOI:** 10.1021/acsanm.3c06052

**Published:** 2024-01-08

**Authors:** Laura Riva, Anna Dotti, Giovanna Iucci, Iole Venditti, Carlo Meneghini, Ilaria Corsi, Ivan Khalakhan, Gloria Nicastro, Carlo Punta, Chiara Battocchio

**Affiliations:** † Department of Chemistry, Materials, and Chemical Engineering “G. Natta”, Politecnico di Milano and INSTM Local Unit, Via Mancinelli 7, 20131 Milano, Italy; ‡ Department of Science, Roma Tre University, Via della Vasca Navale 79, 00146 Rome, Italy; § Department of Physical, Earth and Environmental Sciences, University of Siena, 53100 Siena, Italy; ∥ Department of Surface and Plasma Science, Faculty of Mathematics and Physics, Charles University, V Holešovičkách 2, 18000 Prague, Czech Republic

**Keywords:** nanocomposites, silver-decorated sorbents, *in situ* deposition, water treatment, nanocellulose–heavy metal interactions

## Abstract

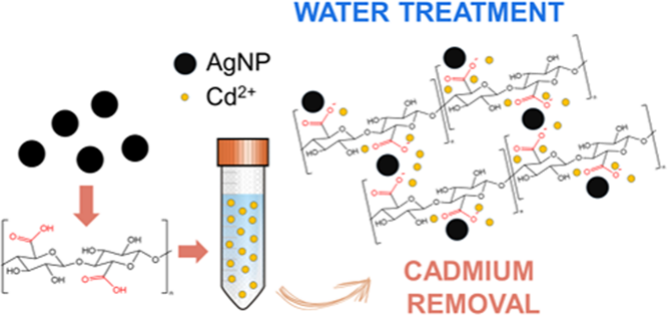

Nanocellulose constitutes a sustainable and biobased solution both as an efficient sorbent material for water treatment and as support for other inorganic nanomaterials with sorbent properties. Herein, we report the synthesis of a nanocomposite by deposition of *in situ*-generated silver nanoparticles (AgNPs) onto TEMPO-oxidized cellulose nanofibers (TOCNFs). Following an in-depth analytical investigation, we unveil for the first time the key role of AgNPs in enhancing the adsorption efficiency of TOCNF toward Cd^2+^ ions, chosen as model heavy metal contaminants. The obtained nanocomposite shows a value of Cd^2+^ sorption capacity at equilibrium from 150 mg L^–1^ ion aqueous solutions of ∼116 mg g^–1^ against the value of 78 mg g^–1^ measured for TOCNF alone. A combination of field emission scanning electron microscopy (FE-SEM), energy-dispersive X-ray (EDX), and X-ray photoelectron spectroscopy (XPS) analyses suggests that Cd^2+^ ions are mainly adsorbed in the neighborhood of AgNPs. However, XPS characterization allows us to conclude that the role of AgNPs relies on increasing the exposure of carboxylic groups with respect to the original TOCNF, suggesting that these groups are still responsible for absorption. In fact, X-ray absorption spectroscopy (XAS) analysis of the Cd–K edge excludes a direct interaction between Ag^0^ and Cd^2+^, supporting the XPS results and confirming the coordination of the latter with carboxyl groups.

## 1 Introduction

Nowadays, manufactured nanomaterials (MNMs), whose structure is characterized by at least one dimension on the order of 1–100 nm,^1^ are applied in a wide range of technological fields, including food processing, advanced therapies, and energy storage.^2,3^

Moreover, in the last two decades, nanotechnology has also emerged as a promising tool for implementing environmental remediation,^4,5^ taking advantage of those unique properties of MNMs, mostly derived from their high surface area, which include a strong chemical affinity, a high surface charge density, and a consequent significant reactivity. In particular, the increasing and rapid deterioration and degradation of the water quality calls for the development of advanced nanosolutions capable of facing the critical issue of effective decontamination, especially focusing on heavy metals, for their known high toxicity for humans and the environment.^6,7^

On the other side, the use of nanotechnology raises concerns in the scientific community and civil society, especially in the field of environmental remediation, due to the potential (eco)toxicity of MNMs, strictly related to the uncertainty about their mobility and transformation.^8,9^ Thus, stakeholders should consider the possible risks associated with the use of MNMs and face them from the very beginning at the design stage, following the guidelines of the eco-design for the synthesis of sustainable and safe solutions.^10^

One of the strategies adopted as the first step for the design of safe MNMs consists of the choice of natural building blocks for their production, possibly derived from waste biomass.^11^ This is why nanocellulose (NC) has attracted high interest in the last two decades as a natural biobased sorbent material for water treatment.^12−15^ Following a bottom-up approach, it is possible to cleave the hierarchical structure of cellulose fibers, leading to the formation of cellulose nanofibers (CNFs) and nanocrystals (CNCs). To enhance the adsorption efficiency of NC toward transition metals, in most cases, further functionalization is necessary for the introduction of either carboxylic^16,17^ or amino groups,^18−21^ capable of better capturing heavy metal ions by electrostatic and/or chelating interaction.

Among the different strategies that can be adopted for CNF production, the regioselective oxidation of the C6 alcoholic groups of the glucopyranose units to the corresponding carboxylic moieties, by means of the 2,2,6,6-tetramethylpiperidinyloxyl (TEMPO)/NaClO/KBr system, is particularly attractive for two distinct reasons. From one side, the chemical modification favors the nanodefibrillation of original fibers by simply moving at basic pH because of the carboxylic group deprotonation and resulting electrostatic repulsion among the negatively charged TEMPO-oxidized nanofibers (TOCNFs).^22^ Moreover, the introduction of carboxylic groups on TOCNF guarantees the electrostatic interaction with heavy metal contaminants, which, combined with the high surface area derived from the nanosize, can provide good adsorption performances.^23−25^ Furthermore, NC can be considered a key building block and support for the synthesis of composites in the presence of inorganic MNMs.^26^

Silver nanoparticles (AgNPs) are undoubtedly among the most promising and applied MNMs in different applications^27^ including water monitoring and treatment.^28^ Mostly due to their antibacterial properties, water sanitation has become an important sector,^29−32^ while other important achievements have also been made in the field of sensing for legacy and emerging contaminants^33,34^ and as photocatalysts for the light-induced oxidative degradation of organic pollutants.^35−37^ Upon proper functionalization, AgNPs have been revealed to be very effective for heavy metal removal from contaminated waters.^38,39^

Nevertheless, despite the great potential and advantages of such applications, AgNP ecotoxicity to both freshwater and marine species has been largely documented.^40−43^ To overcome this issue, a possible solution to limit their mobility and associated toxicity, fixing AgNPs on a suitable support as nanocellulose, might prevent their migration into water and promote a synergic action with the support itself, thus favoring a more efficient decontamination action along with their environmental safety.^44^

Cellulose- and NC-AgNP nanocomposites have already been described and applied in several sectors.^45−48^ In 2018, Ali et al. first reported a cellulose–AgNP composite as an effective sorbent for the removal of metals from aqueous solutions.^49^ More recently, Tavker and co-workers demonstrated how the combination of AgNPs with CNF can provide high adsorption of Cd^2+^ and Cr^3+^ ions.^50^ However, none of these contributions focused on a real comprehension of the reasons for this enhancement in adsorption efficiency.

Herein, we describe for the first time the direct deposition of AgNPs generated *in situ* onto TOCNF and the validation of this new nanocomposite as a sorbent material for the removal of Cd^2+^ ions from aqueous solutions.^18,19,38,50^ Moreover, for the first time, we show how an in-depth physicochemical investigation of the interaction mechanism allows us to unveil the synergistic role of AgNPs in enhancing TOCNF adsorption efficiency by favoring the exposure of the carboxyl groups, suggesting that it is the support (cellulose) and not the nanoparticles that are the active sorbent system.

## 2 Experimental Section

### 2.1 Materials and Equipment

Long-fiber spruce-derived paper was provided by Bartoli Spa paper mill (cellulose content: 95%, fiber length 1–4 mm). All of the other reagents were purchased from Sigma-Aldrich without requiring further purification. Deionized water was produced with a Millipore Elix Deionizer with Progard S2 ion exchange resins. Other equipment used in the procedures include a Branson Sonicator 250 equipped with a 6.5 mm probe tip, a Hanna Instruments HI83141 pH meter, a 220 V 50 Hz laboratory centrifuge, and an SP Scientific BenchTop Pro Lyophilizer.

#### 2.1.1 TEMPO-Oxidized Cellulose Production

TEMPO-oxidized cellulose (TOC) was prepared according to a procedure previously described.^51^ Ten grams of paper derived from long-fiber spruce were minced with the aid of a domestic minipimer in 320 mL of deionized water. Meanwhile, 215 mg of TEMPO and 1.542 g of KBr were dissolved in 250 mL of deionized water (H_2_O_d_) under magnetic stirring at room temperature. Then, a mixture of paper and water was added to the solution. The pH of the obtained mixture was 11.7. Then, 43.7 mL of 12% w v^–1^ NaClO aqueous solution was gradually poured into the mixture, lowering the pH down to 10.2. After that, 5 mL of a 4 M NaOH aqueous solution was added until the pH reached a stable value in the range of 10.5–11.0, indicating that the oxidation process had reached a plateau. After stirring for 18 h at room temperature, 5 mL of a 12 M HCl aqueous solution was added to the mixture to guarantee a final acidic pH, which favored cellulose fibers’ aggregation. Finally, the mixture was filtered on a Büchner funnel and washed with deionized water up to neutral pH. The obtained material was left to dry in air at room temperature for 48 h, providing 7.83 g of dry TOC.

To estimate the oxidation degree of TOC (*i.e*., the mmol of carboxylic groups per gram of material), a titration was performed with a pretitrated NaOH 0.1 N solution, with phenolphthalein as a colorimetric indicator. The TOC oxidation degree was 1.576 mmol_COOH_ g_TOCNF_^–1^.

#### 2.1.2 TOCNF Production

TOCNFs were prepared from TOC according to a procedure previously reported in the literature.^22,52^ Briefly, 5 g of TOC was dispersed in 200 mL of deionized water and an equimolar amount of NaOH with respect to the estimated mmol of −COOH groups was added to the solution (7.88 mmol, 316 mg). The resulting mixture was ultrasonicated with the aid of a Branson Ultrasonifier for 5 min, using an ice bath to control temperature. The mixture was treated with 12 M HCl aqueous solution up to a pH value of 2.00, and it was then filtered under vacuum and washed with deionized water up to neutrality, verified with litmus paper. The recovered wet TOCNF was freeze-dried to completely remove the residual water, obtaining 4.85 g of a white fine powder (yield 97%).

#### 2.1.3 Synthesis of AgNP Composites

**Scheme 1:**
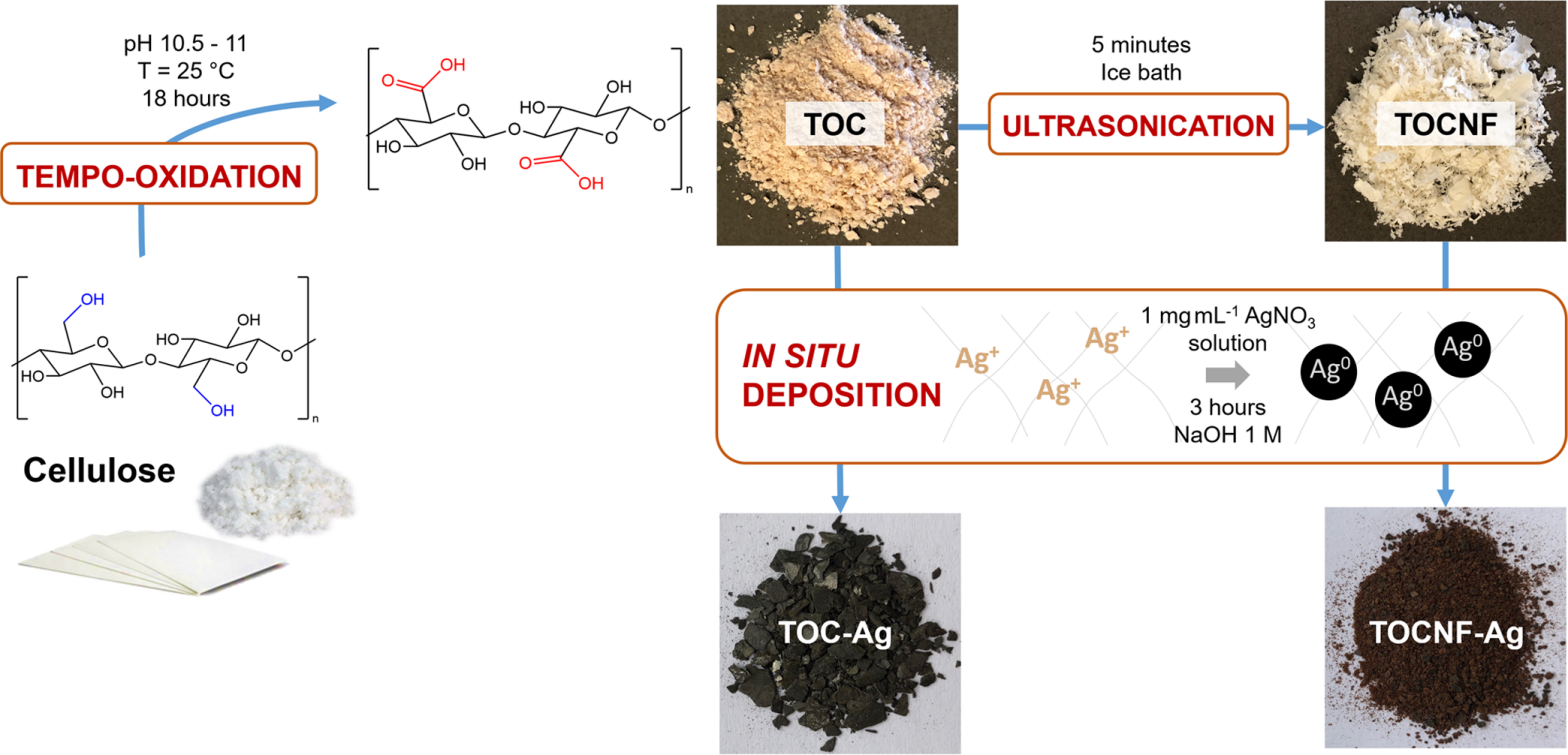
Graphical Description of Synthetic Steps Used for TOC-Ag and TOCNF-Ag Batch Production

Starting from TOC and TOCNF, two new composites (TOC-Ag and TOCNF-Ag) were obtained through the *in situ* deposition of AgNPs. Two g of TOC was ground by means of a mortar and pestle until a fine powder was obtained. The TOC powder was then suspended in 500 mL of H_2_O_d_, together with 500 mg of AgNO_3_. The suspension was stirred at room temperature for 3 h, after which 5 mL of 1 M NaOH aqueous solution was poured into the suspension until its color became black. The suspension was stirred for 1 h. The obtained TOC-Ag was filtered under vacuum and washed with deionized water to reach a neutral pH. The final material was then left to dry in the air for 24 h. The same procedure was followed with TOCNF, leading to the production of TOCNF-Ag (Scheme 1).

#### 2.1.4 Production of NaOH-Conditioned Materials

Neutral materials (TOC, TOCNF, TOC-Ag, and TOCNF-Ag) were conditioned into an alkaline medium to obtain the corresponding deprotonated products (TOC_b_, TOCNF_b_, TOC-Ag_b_, and TOCNF-Ag_b_, respectively). The conditioning procedure, identical for each material, consisted of (i) dispersion of 500 mg of neutral material in a 0,1 M NaOH aqueous solution, (ii) filtration under vacuum, and (iii) washing the filtrate with ethanol. The obtained material was left to dry in air.

### 2.2 Characterization Techniques

Attenuated total internal reflection Fourier transform infrared spectroscopy (FTIR-ATR) was performed using a 640-IR FTIR spectrometer from Agilent Technologies, setting 64 scans for each analysis.

The amount of Ag loaded onto the fibers was determined by inductively coupled plasma optical emission spectroscopy (ICP-OES) analysis on HNO_3_-pretreated solid samples, using a PerkinElmer Optima 3000 SD spectrometer.

Scanning electron microscopy (SEM) was performed by using a variable-pressure instrument (SEM Cambridge Stereoscan 360) at 100/120 Pa with a variable-pressure secondary electron (VPSE) detector. The operating voltage was 20 kV with an electron beam current intensity of 150 pA. The focal distance was 8 mm. The specimens were analyzed in high-vacuum mode after metallization.

The energy-dispersive X-ray spectroscopy (EDS) coupled with SEM allowed us to visualize the silver distribution on the samples’ surface. The analysis was conducted using a Bruker Quantax 200 6/30 instrument.

FE-SEM analysis was conducted by using a MIRA 3 (Tescan) SEM operated with an electron beam energy of 30 keV. The images were collected in backscattered electron (BSE) mode. The element composition of the samples was determined by EDS using an XFlash detector (Bruker) integrated into the SEM.

Transmission electron microscopy (TEM, Philips CM 200, Koninklijke Philips N.V., Amsterdam, Netherlands) analysis, with an acceleration voltage of 200 kV, was conducted on TOC-Ag and TOCNF-Ag samples.

The ζ-potential analyses were conducted with a Malvern Zetasizer Pro-Blue. Each water suspension (0.5 mg mL^–1^) was sonicated in a sonicating bath for 5 min. The suspension was put in the cell and inserted in the zetasizer chamber thermostated at 25 °C. Before the measure, the suspension was left for 60 s to stabilize and then five measurements were performed, waiting 20 s for each measurement. Further information, such as operative pH, is reported in the Supporting Information (SI).

X-ray photoelectron spectroscopy (XPS) data were recorded using a custom-designed spectrometer, described in previous studies^53^ and equipped with a nonmonochromatized Mg Ka X-ray source (1253.6 eV pass energy 25 eV, step 0.1 eV). For this experiment, photoelectrons emitted by C 1s, O 1s, Ag 3d, Cd 3d, and Na 1s core levels were detected on TOCNF powder (solid-state) samples either pristine or with included AgNPs prepared *in situ* (see Section 2.1.3) and exposed to Cd^2+^ ions. All spectra were energy-referenced to the C 1s signal of aliphatic C atoms having a binding energy (BE) of 285.00 eV.^54^ Atomic ratios were calculated from peak intensities using Scofield’s cross-sectional values.^55^ Curve-fitting analysis was performed using Gaussian profiles as fitting functions after subtraction of a polynomial background. For qualitative data, the BE values were referred to the NIST database.^56^

X-ray absorption spectroscopy (XAS) experiments were carried out at the LISA (BM08) beamline at ESRF (European Synchrotron Radiation Facility, Grenoble, France),^57^ (CERIC–ERIC proposal #20210006). The TOCNF powders containing AgNPs and exposed to Cd^2+^ ions were hand-grinded and pressed (5Ton) to obtain thin solid homogeneous pellets suitable for handling. The Ag–K and Cd–K edge XAS spectra were collected at room temperature in fluorescence mode using an ultrapure 13 element Ge multidetector. The Ag or Cd *Kα* fluorescence signals were electronically selected out of the total fluorescence yield, using the multichannel analyzers associated with each detector element. The death time for each detector channel was kept below 5% to avoid nonlinearity effects. The XAS spectra measured from pure metal foils (Ag or Cd) placed along the X-ray beam after the sample are used for precise X-ray beam energy calibration. The XAS signal (α) was calculated by summing up the fluorescence signals from each detector *I*_*i*_
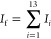
1and normalizing by the incoming X-ray beam intensity (*I*_o_) measured using an Ar-filled ionization chamber
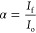
2The raw XAS signal has been treated along the standard procedures^58^ to extract the structural XAFS signal χ(*k*) in which the photoelectron wavenumber *k* is defined as
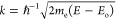
3*m*_e_ being the electron mass, *E* being the X-ray beam energy, and *E*_o_ being the edge energy selected at the first inflection point of the absorption edge and refined during the data analysis.

### 2.3 Procedure for Sorption Experiments

Sorption tests in dynamic conditions were performed using a Heldolph multi reax shaker set at 450 rpm. Cd^2+^ concentration in aqueous solutions was determined through ICP-OES using a PerkinElmer Optima 3000 SD spectrometer, with a limit of detection (LOD) and a limit of quantification (LOQ) equal to 0.005 mg L^–1^ for cadmium. Calibration was done at 50–100–150 mg L^–1^, and the analyses were conducted on three spectral lines. Twelve mg (±0.2 mg) of the material under investigation was put into a Falcon vial and dispersed in 15 mL of monocontaminated Cd^2+^ 150 mg L^–1^ aqueous solution, prepared by dissolving 312.6 mg of CdCl_2_ in 1 L of H_2_O_d_. The vials were shaken for 24 h at room temperature by means of an orbital shaker, after which the solutions were filtered on filter paper. Metal ion concentrations were determined by ICP-OES analysis of the solution. The sorption capacity at equilibrium *Q*_e_ (mg g^–1^) was used as a descriptive parameter to compare materials in the discontinuous adsorption tests we conducted. Once an initial concentration *C*_0_ is chosen, during a discontinuous batch adsorption operation, the adsorbate is transferred to the adsorbent surface, decreasing its concentration in the solution until the final value of *C*_e_ and increasing its quantity in the solid phase until *Q*_e_. A simple mathematic treatment is normally performed to obtain the amount of adsorbate adsorbed into the adsorbent at equilibrium (*Q*_e_), following eq 4
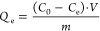
4where *V* is the solution volume (L), while m indicates the mass of sorbent material (g).

The same experiments were repeated also on solutions containing different concentrations of interfering cations, namely, Na^+^ and Ca^2+^. To assess this aspect, Cd^2+^ 150 mg L^–1^ solutions containing 100 and 10000 mg L^–1^ of Na^+^ concentration and 100 mg L^–1^ of Ca^2+^ concentration were prepared by dissolving 312.6 mg of CdCl_2_ in 1 L of water containing 254 mg of NaCl (for the 100 mg L^–1^ Na^+^ solution), 1 L of water containing 25.4 g of NaCl (for the 10,000 mg L^–1^ Na^+^ solution), and 1 L of water containing 276.9 mg of CaCl_2_ (for the 100 mg L^–1^ Ca^2+^ solution). Sorption experiments for these solutions were conducted as reported above, and an experiment was also conducted at 72 h of sorbent-solution contact to verify the achievement of sorption equilibrium.

## 3 Results and Discussion

### 3.1 Synthesis and Characterization

As previously reported, the regioselective oxidation pathway to TOC and TOCNF production carries with it the advantage of obtaining a prefunctionalized and overoxygenated surface. These characteristics guaranteed the ideal environment for the *in situ* generation of AgNPs from AgNO_3_ and their consequent deposition on the substrate by simply taking advantage of the reducing efficiency of the same cellulosic support. Indeed, in line with what has been already reported for other CNC/AgNP composites, no additional reducing agents were necessary, while the presence of NaOH was required from a kinetic point of view, as it has the function of speeding up the process by promoting the formation of AgOH and Ag_2_O intermediates.^59^

Products were first qualitatively characterized by FTIR-ATR analysis, and the recorded spectra for TOC-Ag, TOCNF-Ag, TOCNF, and TOCNF_b_ samples are shown in Figure S1 in the SI. Considering TOCNF, it is possible to observe the peak at 1725 cm^–1^, which is associated with the C=O stretching of the carboxyl group COOH, thus confirming the regioselective conversion of the C6 alcoholic group by TEMPO-mediated oxidation. As expected, for TOCNF_b_, obtained by the alkaline treatment of TOCNF, the same C=O stretching signal shifts at 1600 cm^–1^ due to the corresponding carboxylate anion −COO^–^. Interestingly, by comparing the spectra of AgNP-decorated samples, we registered the presence of two additional peaks at about 2700 and 2900 cm^–1^, which are characteristic of the C–H stretching of the H–CO aldehydic groups. This evidence confirms the active role of TOCNF in reducing Ag^+^ ions, probably also by means of the introduced carboxylic groups, which, however, are still present in both the TOC-Ag and TOCNF-Ag products. Moreover, the stretching band of the OH group between 3200 and 3500 disappears after AgNP-loading in TOC and TOCNF, confirming that the interaction and fixing of nanoparticles on the cellulosic structure highly involve the alcoholic groups of the glucopyranose units, as also reported for other nanocellulose-based composites.^48^

ICP-OES analyses conducted on TOC-Ag and TOCNF-Ag allowed us to quantify the silver amount on composite materials. The loading, expressed in wt % of Ag, seems to be slightly lower for TOC-Ag (4.50 ± 0.02 wt %) than for TOCNF-Ag (6.92 ± 0.02 wt %), probably because of the higher exposed surface of nanofibers.

**Figure 1:**
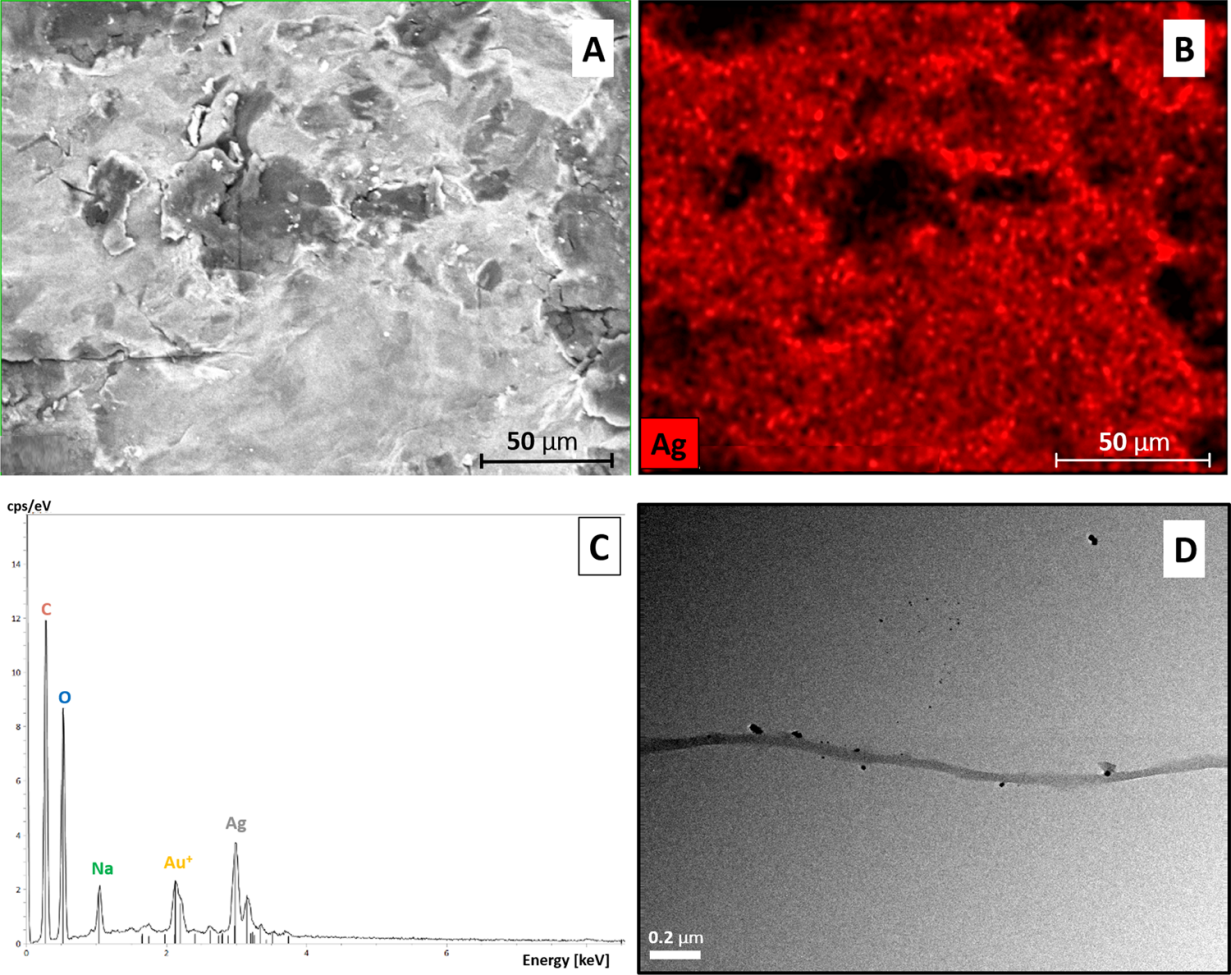
(A) SEM image of TOCNF-Ag; (B) EDS visual characterization of TOCNF-Ag, with silver distribution highlighted in red; (C) EDS spectrum for TOCNF-Ag; and (D) TEM images of TOCNF-Ag.

Moreover, SEM-EDS analyses confirmed a homogeneous distribution of Ag on the surface of TOCNF (Figure 1), as highlighted by the red color (Figure 1B), while the presence of Na should be ascribed to the NaOH used in the synthetic procedure (Figure 1C).

Finally, TEM images of TOCNF-Ag clearly showed the deposition of AgNPs on the nanofibers (Figure 1D), confirming the effectiveness of the synthetic process and the achievement of the desired composite. No aggregation of AgNPs was observed on the nanofibers’ surface. This observation is in line with what was reported by Valencia et al. for the *in situ* growth of metal-oxide nanoparticles, including Ag_2_O, on TOCNF.^60^ In fact, in that case, the authors also emphasized the role of nanofibers in preventing the coagulation of nanoparticles in larger clusters.

As a further characterization, **ζ**-potential measurements were conducted on the samples. Even if the measurements were not conducted on spheres, but on fibers, so that they cannot be related to DLS-size analysis, they provide a qualitative but clear trend of the **ζ-**potentia, confirming what has been recently reported in the literature:^61^ a negative value is observed due to the introduction of carboxylic groups on the nanofiber backbone (Table S2, entry 3 in the SI). Surprisingly, the loading of AgNPs further promotes the decrease of the ζ-potential, suggesting an increment of negative charges on the surface of the resulting nanocomposite (Table S2, entry 6 in the SI). On the contrary, this value increases after Cd^2+^ adsorption on the system (Table S2, entry 7 in the SI).

### 3.2 Sorption Experiments

**Figure 2:**
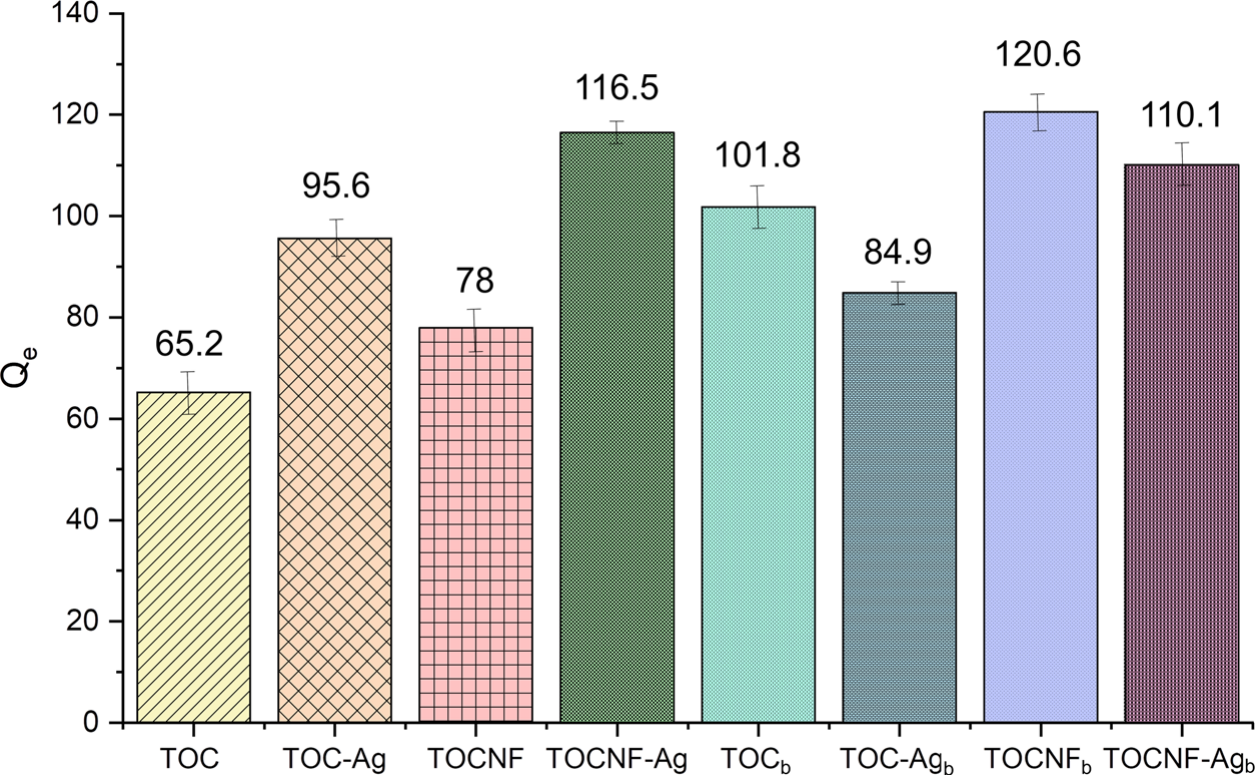
Scheme of adsorption tests conducted with neutral and alkaline materials in 150 mg L^–1^ of Cd^2+^ solutions.

Cd^2+^ sorption tests were carried out to verify and compare the sorption properties of the synthesized materials. Preliminary experiments were performed in the presence of TOC, TOC-Ag, TOCNF, and TOCNF-Ag, following the experimental conditions described in Section 2.3. Results are reported in Figure 2 and in the Supporting Information (see Table S3, entries 1–4), from which two different trends can be highlighted.

First, Cd^2+^ sorption capacity increases when moving from TOC to TOCNF, a nanosized sorbent material, and this is true both in the absence (Table S3, entry 1 compared to entry 3) and in the presence of silver nanoparticles (Table S3, entry 2 compared to entry 4). Moreover, AgNP decoration determines a significant improvement of Cd^2+^ removal capacity for both TOC and TOCNF (Table S3, entries 2 and 4 compared with entries 1 and 3, respectively).

To better investigate the role of carboxylic groups in Cd^2+^ capture efficiency, the same sorption experiments were repeated using the same four materials pretreated under alkaline conditions to obtain TOC_b_, TOC-Ag_b_, TOCNF_b_, and TOCNF-Ag_b_.

As can be detected in Figure 2 and Table S3 (entries 5–8), the material’s treatment with an alkaline solution strongly increases the average adsorption capacity of TOC and TOCNF, resulting in *Q*_e_ values for TOC_b_ and TOCNF_b_ (Table S3, entries 5 and 7) as high as those of TOC-Ag and TOCNF-Ag (Table S3, entries 2 and 4). On the contrary, basic pretreatment of AgNP-decorated materials does not significantly affect the sorption performance, with just a slight decrease in *Q*_e_ values (Table S3, entries 6 and 8).

It is worth highlighting here how TOCNF_b_, even though performing as well as TOCNF-Ag, is interesting to be investigated solely to better understand the interaction mechanism with Cd^2+^, as for a decontamination process point of view, they are highly dispersed in the medium and consequently difficult to be processed and recovered after water treatment. On the contrary, both TOCNF and TOCNF-Ag can be easily recovered by filtration.

### 3.3 Interaction Mechanism

With the aim to gather insights into the mechanism of interaction of TOCNF with AgNPs and Cd^2+^ ions, pristine TOCNF, TOCNF-Ag, and TOCNF-Ag + Cd samples were investigated, combining FE-SEM, XPS, and XAS. Such a multiphysics approach allowed us to define a reliable model for the TOCNF–metal interaction that accounts for the increased ability of TOCNF-Ag to absorb Cd^2+^ compared to pristine TOCNF, as reported in Section 3.2.

**Figure 3:**
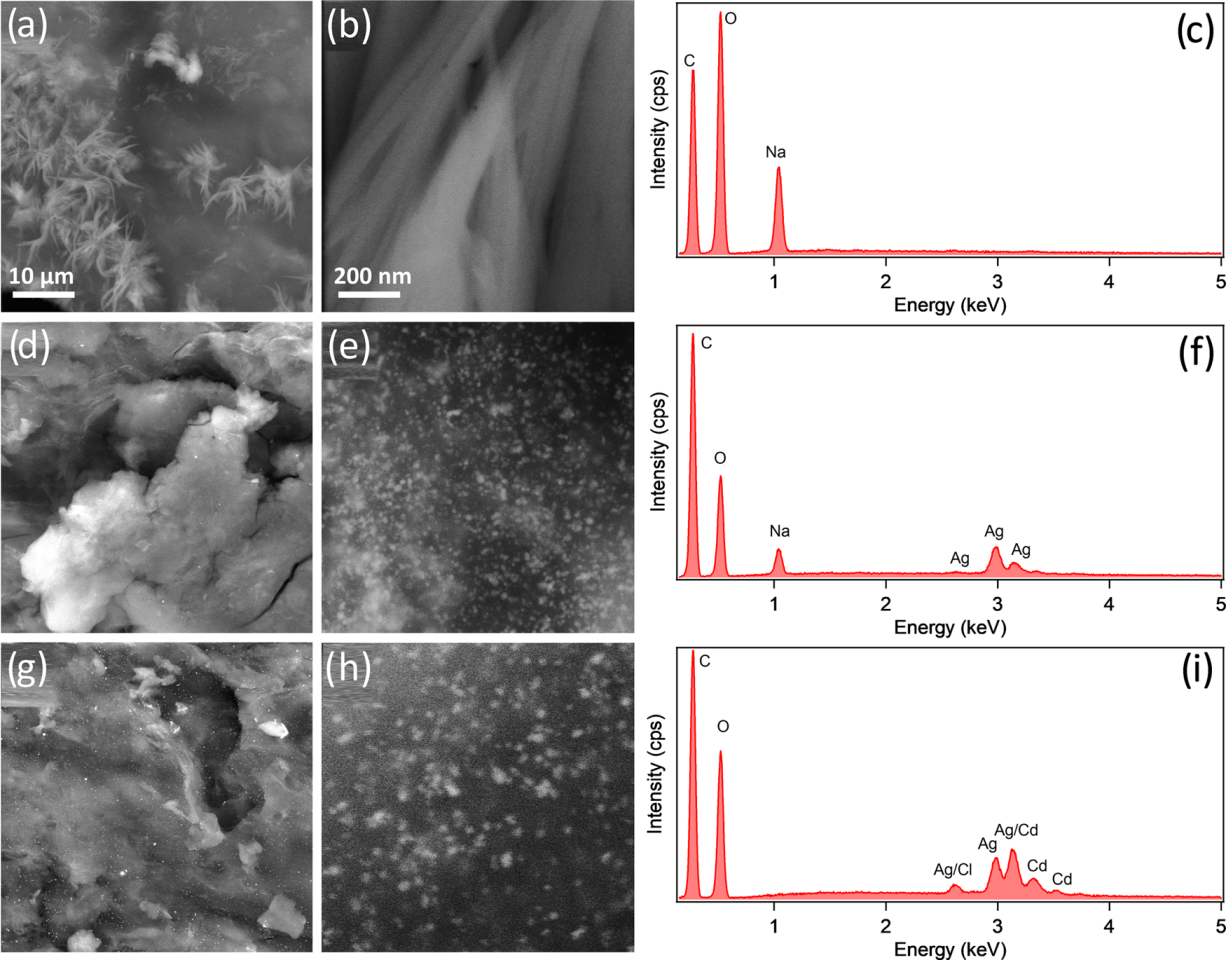
FE-SEM images and EDX profiles acquired on TOCNF (a–c), TOCNF-Ag (d–f), and TOCNF-Ag + Cd (g–i).

The SEM images and corresponding EDX spectra are summarized in Figure 3. The SEM in Figure 3a,3b reveals the presence of fibers in the TOCNF sample as well as the expected signals of oxygen, carbon, and sodium emerging on the EDX spectrum in Figure 3c. However, when TOCNF is combined with AgNPs, one can observe a change in the shape of the fibers and the emergence of homogeneously distributed small AgNPs with sizes ranging between 5 and 10 nm (Figure 3d,e). EDX analysis in Figure 3f confirms that oxygen, carbon, sodium, and silver are present in this material. When the TOCNF-Ag sample is exposed to Cd^2+^ ions, the AgNPs become evidently larger, ranging between 15 and 20 nm as shown in Figure 3g,h. Some larger aggregates can also be observed in the corresponding SEM image. EDX analysis of this material shown in Figure 3i clearly shows the presence of both Ag and Cd, suggesting that the Cd^2+^ ion adsorption is localized near silver nanoparticles. It is also noteworthy that the presence of Na is no longer observed. However, some traces of chlorine appear in the EDX spectrum.

XPS measurements were carried out at C 1s, O 1s, Ag 3d, and Cd 3d core levels on pristine TOCNF, TOCNF-Ag, and TOCNF-Ag + Cd. TOCNF_b_, TOCNF_b_-Ag, and TOCNF_b_-Ag + Cd were also investigated for comparison due to the high affinity of TOCNF_b_ for Cd^2+^ (see Section 3.2). A complete collection of XPS data analysis results (binding energy, full width half-maxima, atomic percentage values, and proposed assignments) is reported in Table S4 in the Supporting Information. Here, the most interesting results will be summarized and discussed.

**Figure 4:**
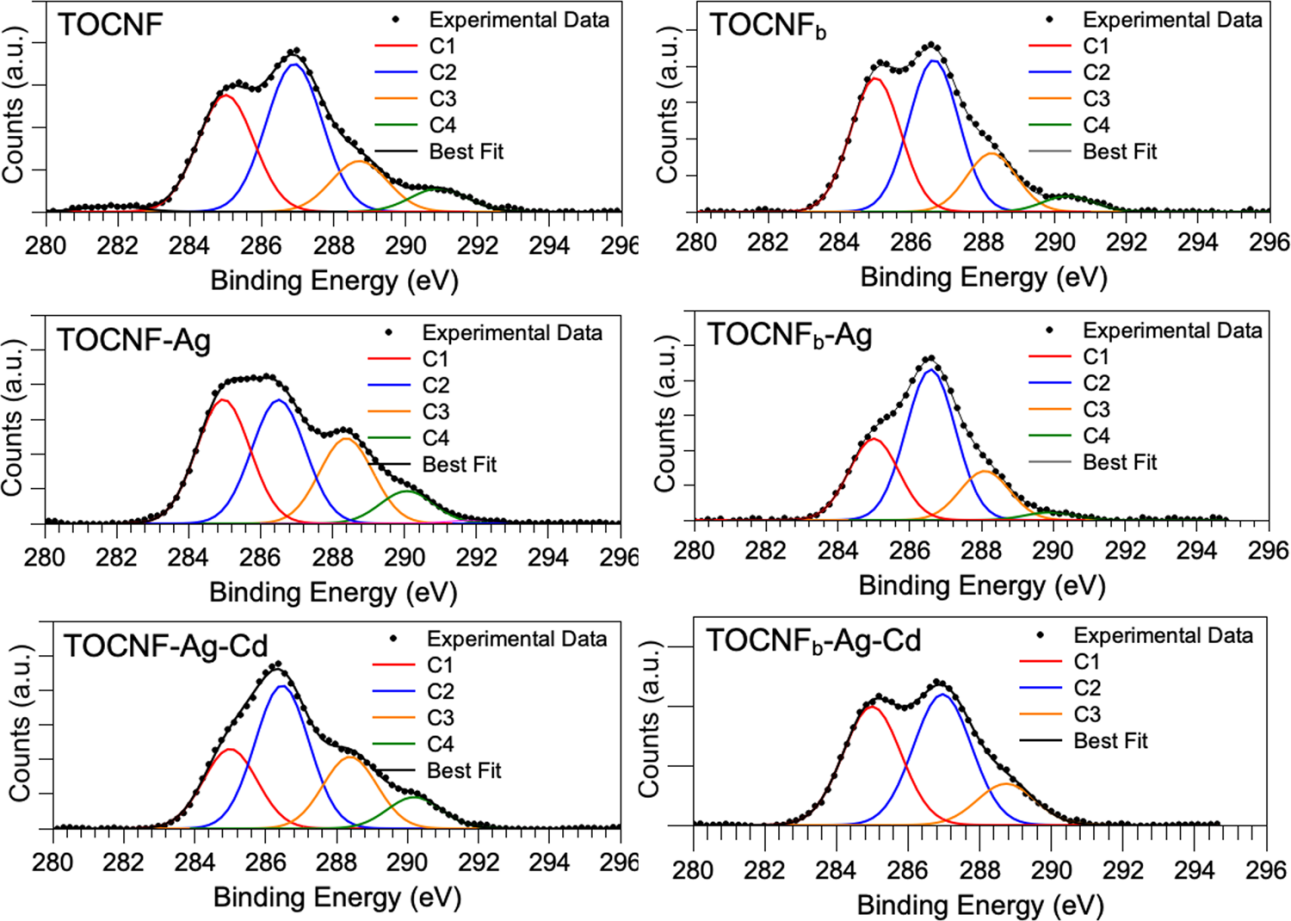
XPS C 1s spectra of TOCNF, TOCNF-Ag, and TOCNF-Ag + Cd (on the left) and TOCNF_b_, TOCNF_b_-Ag, and TOCNF_b_-Ag + Cd (on the right).

C 1s spectra (Figure 4) appear composite for all samples; by applying a curve-fitting procedure, it is possible to individuate four spectral components, coherently with literature findings for oxidized cellulose.^62−65^ The BE values observed for the four spectral components (namely, C1–C2–C3–C4) are in excellent agreement with literature reports and highly reproducible upon TOCNF enrichment with AgNPs and Cd^2+^ absorption, confirming the overall stability of the TOCNF chemical and electronic structure. The first component at lower BE values (C1, BE = 285.00 eV) is associated with C–C and C–H and usually chosen for calibration purposes (see Section 2); the peak at 286.9 eV (C2) is attributed to C–O groups (both C–OH and C–O–C). For the assignment of components C3 and C4, respectively, at about 288.5 and 290 eV, there is some debate in the literature. While it is expected that O–C–O carbon atoms have a C 1s BE of about 288.5 (C3), the carboxylate functional groups COO^–^ are either reported superimposed to O–C–O (C3 = O–C–O + COO^–^)^62^ or at higher BE^65^ (C3 = O–C–O; C4 = COO^–^ + COOH). On the other hand, COOH carbon atoms are usually found around 290 eV (C4). For clarity, the two possible assignments (namely, “A” and “B”) are summarized in Table 1.

**Table 1:** Proposed Assignments for C 1s Spectral Components

(A)	(B)
C1 = C–C	C1 = C–C
C2 = C–OH, C–O–C C3 = O–C–O, COO^–^	C2 = C–OH, C–O–C
C4 = COOH	C3 = O–C–O
	C4 = COO^–^, COOH

From C 1s spectra reported in Figure 4, it is noteworthy that the TOCNF sample prepared at pH = 12 and exposed to Cd^2+^ (TOCNF_b_-Ag + Cd) does not show the C4 component; what is more, all TOCNF_b_ systems have a C4 component of very low intensity. This can indicate that the correct assignment, for our nanostructured oxidized cellulose, is the “A” scheme since the C4 component corresponding to the COOH groups is expected to decrease or disappear due to deprotonation. In addition, the semiquantitative analysis reported in Table S4 in the Supporting Information shows better correspondence between the experimental atomic percentages and the theoretical values calculated for the (A) scheme than for the (B) one; the consistency between experimentally calculated and theoretically estimated atomic percentage values in all samples (except for the already discussed C4 component in TOCNF_b_-Ag + Cd) also confirms the TOCNF molecular structure stability upon enrichment with AgNPs and adsorption of Cd^2+^ ions. Going into a deeper semiquantitative data analysis, in Table 2, the atomic percentages of the C3 and C4 (C3/C3 + C4; C4/C3 + C4) components are reported as calculated from the peak-fitting procedure.

**Table 2:** Atomic Percentages Calculated for C3 and C4 Components over C3 + C4^a^,^66^

assignment (A)	TOCNF	TOCNF-Ag	TOCNF-Ag + Cd	TOCNF_b_	TOCNF_b_-Ag	TOCNF_b_-Ag + Cd
O–C–O, COO^–^ (C3)	68.5	60.1	69.6	78.8	86.5	100
COOH (C4)	31.5	39.9	30.4	21.2	13.5	

a The statistical incertitude in the semiquantitative evaluation by XPS is estimated as 5% of the calculated value.

As the first observation, the decoration of TOCNF with AgNPs leads to an increase of the C4 contribution, which can indicate a higher exposition of carboxylic groups on the surface. This result is in line with what was observed by measuring the ζ**-**potential of nanocomposites, with an increase of the negative charge moving from TOCNF to TOCNF-Ag (Table S2 in the SI). By considering that this trend is accompanied by an increase in the adsorption performance for TOCNF-Ag, we can assume that the higher exposition of the carboxylic groups should be directly related to Cd^2+^ adsorption. Apparently opposite, the trend observed for TOCNF_b_ when compared with that of TOCNF_b_-Ag can somehow confirm the role of AgNPs. In fact, in this case, we should assume that the COO^–^ contribution is obviously higher in TOCNF_b_, as TOCNF has been pretreated in an alkaline environment. Under these conditions, a higher exposition of carboxylic groups derived from AgNP decoration results in a higher contribution of C3 over C4, in line with the more negative value of ζ-potential previously discussed for AgNPs. Moreover, the C3 and C4 atomic percentages also suggest that, going from TOCNF-Ag to TOCNF–Ag-Cd samples, the relative amount of COO^–^ groups increases with respect to the COOH, and this effect is observed for both TOCNF and TOCNF_b_ systems. Such negatively charged residues can be responsible for the efficient interaction with the positively charged Cd^2+^ ions, as will be explained in the following.

**Figure 5:**
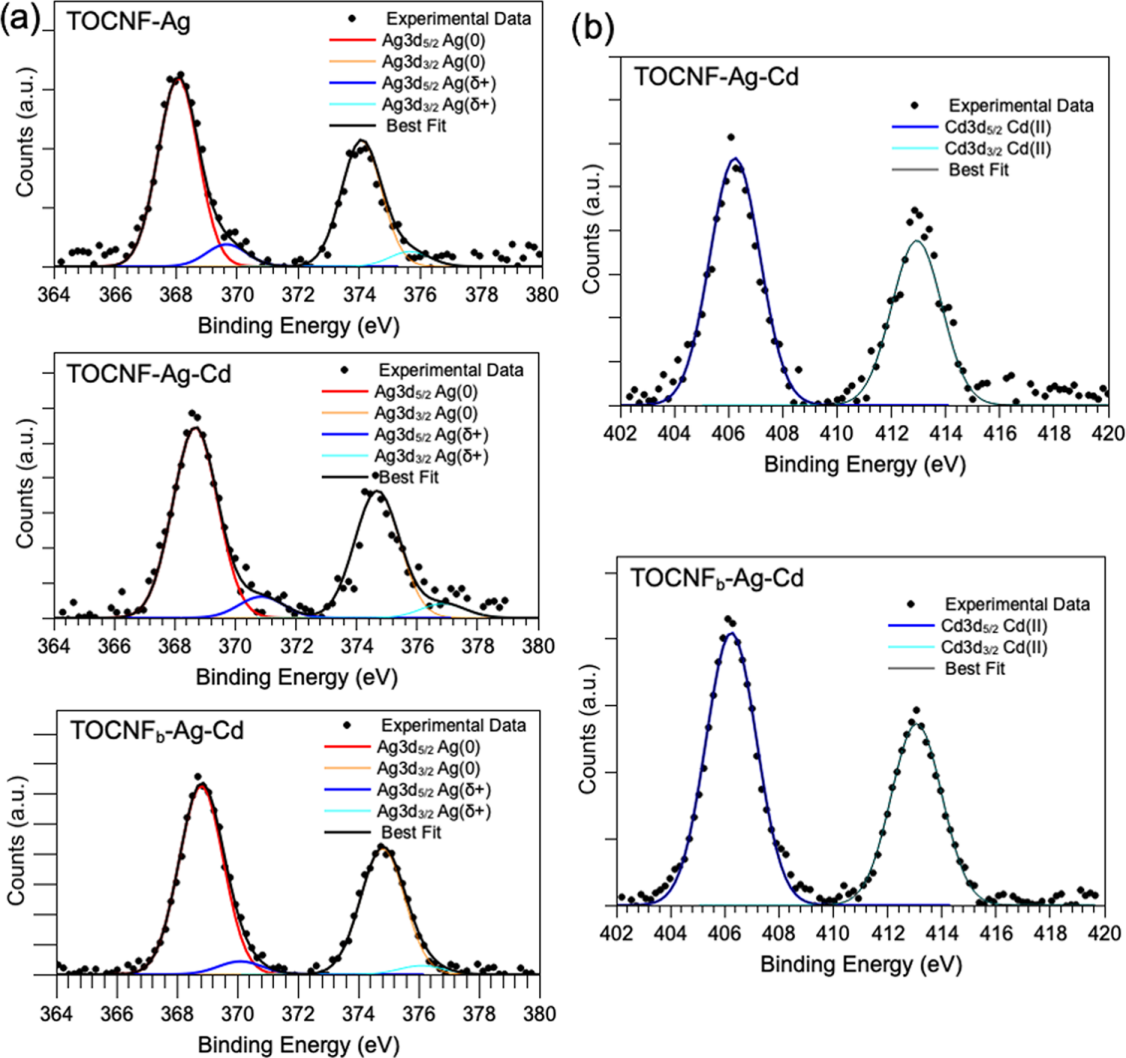
XPS spectra of Ag 3d (a) and Cd 3d (b) acquired on TOCNF-Ag, TOCNF-Ag+Cd, and TOCNF_b_-Ag + Cd.

In Figure 5, the XPS spectra of Ag 3d (a) and Cd 3d (b) acquired on the samples exposed to metals are reported. For Cd 3d, a single spin–orbit pair is always observed and attributed to Cd^2+^ ions (Cd 3d_5/2_ BE = 406.24 eV).^56^ Ag 3d spectra, on the other hand, are composite, and by applying a peak-fitting procedure, it is possible to individuate two spin–orbit pairs in all samples. The main signal at lower BE (Ag 3d_5/2_ BE at about 368 eV) is assigned to metallic silver atoms at the NP core; the low-intensity features at higher BE values (Ag 3d_5/2_ BE around 370 eV) are due to partially positively charged silver ions at the NP surface, coherently with the literature;^67^ it is noteworthy that these Ag^δ+^ atoms are expected by the literature to interact with capping agents at the NP surface;^67^ here, the envisaged partners for such interactions are the carboxylate groups of TOCNF.

To gather detailed information about the local structure of Ag and Cd atoms, XAS measurements were carried out on the sample TOCNF–Ag-Cd. Data were collected in both the near-edge (XANES) and extended (EXAFS) regions at Ag and Cd K edges.

**Figure 6:**
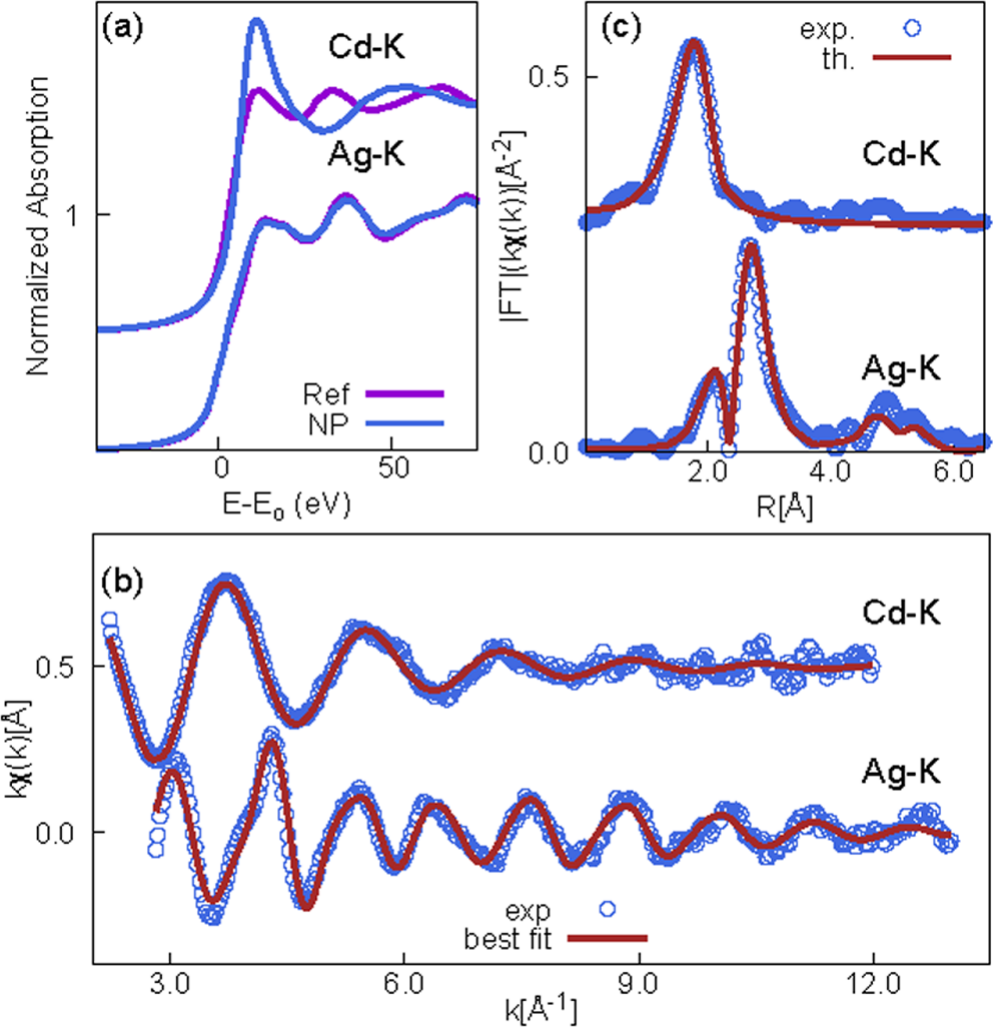
Ag and Cd K edge XAFS results on the TOCNF-Ag + Cd sample: (a) XANES region of the sample and reference metal foils; (b) experimental data (circles) and best fits (dark red lines) for Ag and Cd K edges; and (c) module of the Fourier transform of experimental data (blue circles) and best fit (dark red lines).

The normalized Ag–K and Cd–K edge XAS spectra in the XANES regions are reported in Figure 6a, along with the reference spectra measured on pure Ag and Cd metal foils for the sake of comparison. The Ag XANES spectrum measured in the TOCNF-Ag + Cd sample matches well the Ag XANES spectrum measured on the reference metal, suggesting that most of the Ag atoms in this sample reside in the relatively large nanoparticle bulk. Instead, the Cd XANES spectrum is definitively different from that of the Cd metal foil: the high energy edge shift and the intense white-line at the edge suggest that Cd atoms are oxidized in excellent agreement with XPS Cd 3d spectra, revealing Cd^2+^ ions.

The quantitative analysis was performed by fitting the k-weighted experimental EXAFS spectra *kχ*(*k*) to the theoretical curves calculated using the standard EXAFS model^68^ assuming Gaussian disorder. The photoelectron amplitude and scattering functions were calculated using the FEFF8 program^65^ and the silver bulk atomic structure model. The Ag–K edge spectrum was fitted in the 3–13 Å^–1^, selecting the relevant single scattering, and the selected multiple scattering contributions were used to take into account the main structural features until about 5.8 Å from the average absorber; constraints based on the Ag bulk crystallographic structure were applied in order to reduce the number of free parameters along the lines described in Battocchio et al.^69^ The Ag EXAFS spectra *kχ*(*k*), and the best-fit model are shown in Figure 6a for the sake of comparison. In the XANES region (panel a of Figure 6), the normalized XAS spectra measured for the sample and reference metal foils are shown for comparison; the energy scale is presented with respect to the edge energy (*E*–*E*_o_), and the Cd data are vertically shifted for the sake of clarity. The structure of metal Ag allows well accounting for the main structural features of Ag in our samples, confirming that most of the silver atoms in the sample belong to a bulk Ag phase as expected for relatively large nanoparticles without a sizable Ag dispersion. The lattice parameter of Ag in our sample appears slightly compressed (2%) with respect to metallic Ag, which is an effect typical in NPs.

The Cd EXAFS features appear much smoother (Figure 6b) with respect to Ag data, with a single main peak around 2 Å in the Fourier transform (Figure 6c), pointing out an averagely much more disordered environment around Cd with respect to Ag. Noticeably, in the near-edge region, the effect of the structural disorder is weak; therefore, the XANES features can be considered as the fingerprint of the specific coordination environment of the absorber in the samples. Comparing the experimental Cd XANES measured in TOCNF-Ag + Cd and literature data, we found a close similarity with the spectrum of Cd-acetate Cd(CH_3_COO)_2_, suggesting that the Cd^2+^ state in our sample directly interacts with COO^–^ groups revealed by XPS analysis.^70^ This is also in agreement with the EDX analysis reported in Figure 3i, showing that Na is not observed in the presence of Cd, thus suggesting that Cd^2+^ ions substitute for Na^+^ ions in the electrostatic interaction with the carboxylate groups.

In the analysis, we found about 6 oxygen neighbors at an average distance of 2.29(1) Å around the Cd absorber, with a mean square relative displacement for the Cd–O shell equal to σ^2^ (CdO) = 7.8(3) x 10^–3^ Å^2^. Attempts to include in the analysis the next neighbor shells, which can provide deeper details about the Cd coordination chemistry, did not statistically improve the refinement. Noticeably, the Ag and Cd have very similar backscattering amplitude and phase functions; therefore, they cannot be distinguished from the EXAFS analysis. However, the photoelectron amplitude and phase functions of Ag and Cd are clearly different from that of oxygen and can be easily distinguished; therefore, the absence of a significant signal from a heavy neighbor shell such as Cd–Ag(Cd) allows the exclusion of a direct interaction between the Ag nanoparticles and the Cd ions.

Summarizing FE-SEM, XPS, and XAS data analysis results, FE-SEM images point out the presence of larger aggregates in TOCNF-Ag + Cd, while EDX spectra suggest the presence of both Ag and Cd in the larger NPs but not Na (conversely found in TOCNF and TOCNF-Ag). XPS investigation evidences a higher degree of deprotonation of COOH functional groups in the presence of Cd^2+^, suggesting that Cd ions can electrostatically interact with the carboxylate moieties of TOCNF in the neighborhood of AgNPs. Such a hypothesis is confirmed by XAS analysis, which, on the one hand, reveals that Cd^2+^ only coordinated with the COO^–^ groups and, on the other, excludes the direct interaction between Ag atoms and Cd ions.

### 3.4 Sorption Experiments in the Presence of Na^+^ and Ca^2+^

To better confirm the electrostatic nature of the sorbent–sorbate interaction and the contribution of the coordination interactions observed by XAS analyses, Cd^2+^ sorption capacity was evaluated in the presence of a competitive monovalent cation (Na^+^) at two different concentrations (100 mg L^–1^ and 10 g L^–1^) and a bivalent cation (Ca^2+^) at a 100 mg L^–1^ concentration. These tests were carried out only on TOCNF, TOCNF-Ag, and TOCNF_b_, which were the materials showing the best sorption performances. The first experiment was conducted by fixing a sorbent/solution contact time of 24 h.

**Figure 7:**
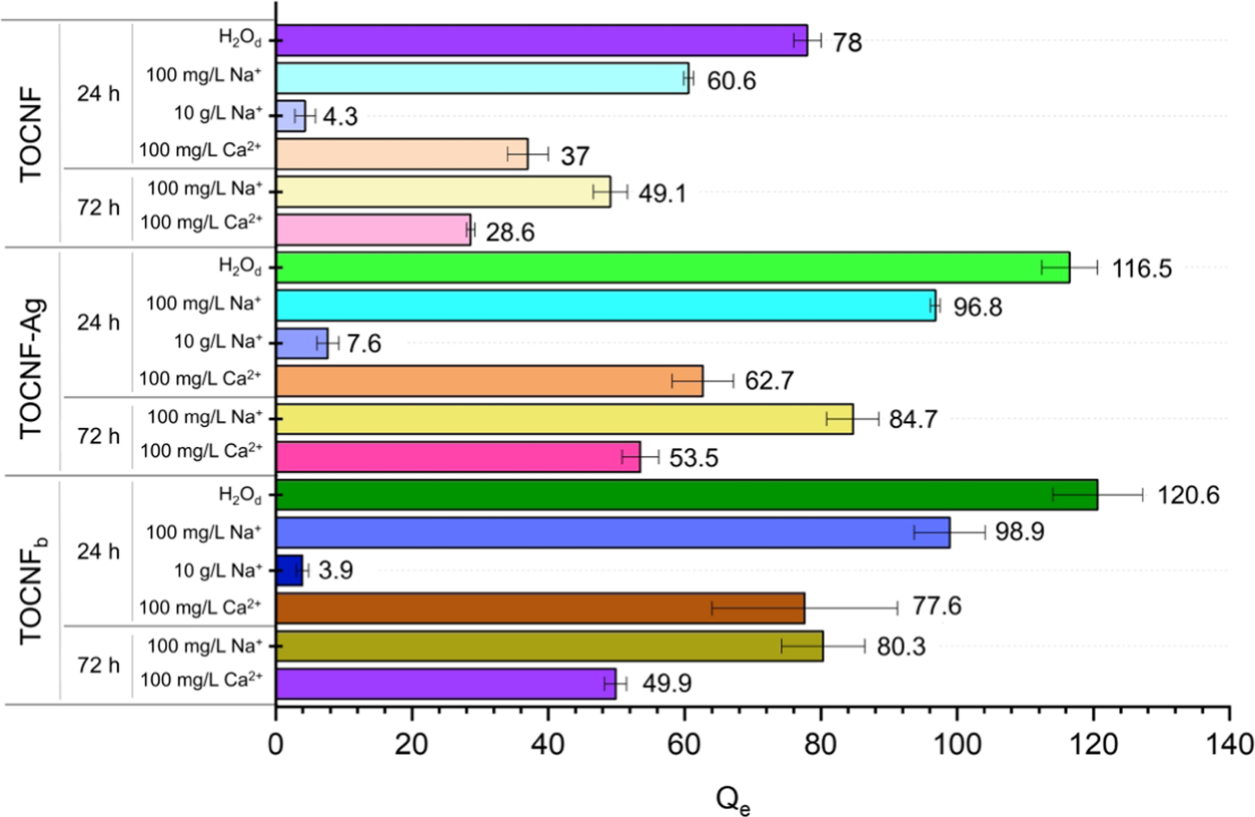
Results of the adsorption experiments conducted in the presence of interfering cations (comparison between 24 and 72 h).

The collected data, depicted in Figure 7 and Table S5, show that Na^+^ ions hinder the interaction between – COO^–^ groups and Cd^2+^. This fact is evident as the materials’ sorption capacity is gradually reduced with the increment of Na^+^ concentration (from 100 mg L^–1^ to 10000 mg L^–1^). In the presence of bivalent positive ions Ca^2+^ (100 mg L^–1^ concentration), it is also possible to observe a decrement of the adsorption properties of the materials, suggesting a shielding activity by Ca^2+^ as well. Comparing the two interfering ions, Na^+^ and Ca^2+^, at the same concentration (100 mg L^–1^), it can also be seen that the shielding activity for the bivalent cation is higher than that for the monovalent cation.

By repeating the experiment using 72 h as the sorbent-solution contact time and comparing the results with those obtained after 24 h, it can be observed that the same trend is maintained. It can be noticed that the adsorption capacity of the composite is further reduced by prolonging the time of the experiment, thus emphasizing the shielding role of the bivalent Ca^2+^ ion.

These experiments in the presence of interfering ions thus confirmed the crucial role of the ionic interaction between adsorbent materials and Cd^2+^ ion, further suggesting the presence of chelation bonds between the adsorbent and adsorbate, which are more shielded in the presence of interfering ions of similar charge and size to Cd^2+^. Nevertheless, it is worth noting that only when operating at very high concentrations of Na^+^ or Ca^2+^ (10 g L^–1^) the adsorbent effect of TOCNF_b_ and TOCNF-Ag is suppressed, while at lower but more realistic concentrations (100 mg L^–1^), both the systems are highly selective toward the transition-metal ion.

## 4 Conclusions

MNM safety is becoming mandatory along with their efficacy in environmental applications such as remediation. Nanocomposites are seen as promising in enhancing single MNM properties but more important in limiting NP mobility and associated ecotoxicity. Here, we have reported an in-depth investigation on the synergic role of AgNPs loaded on TOCNF in promoting and enhancing the adsorption of Cd^2+^ ions from aqueous solutions. AgNPs were generated *in situ* under alkaline conditions thanks to the reducing action of TOCNF and resulted in homogeneously fixed on the nanofiber backbone. While TOCNF had been proposed as a suitable support for AgNPs, which were expected to play the main role in interacting with Cd^2+^ ions, the combination of data analysis results provided a different interpretation. In fact, the loading of AgNPs on the nanofiber led to an increase of the exposed carboxylic groups, as supported by XPS analysis. The same carboxylic moieties are found to be the main responsibility for Cd^2+^ adsorption, with an increase of COO^–^ contribution for TOCNF-Ag + Cd samples, suggesting an electrostatic interaction between the negatively charged composite and the heavy metal cations. Importantly, while FE-SEM images and EDX show that this interaction occurred in the neighborhood of AgNPs, both XPS and XAS data coherently exclude some direct interaction between Ag^0^ and Cd^2+^ ions, showing that Cd^2+^ adsorption is mainly related to the direct Cd^2+^–COO^–^ interaction. This finding once again emphasizes the relevance of the multiphysics approach to the study of these complex nanostructured materials.

## Supporting Information Available

FTIR-ATR analysis; values of pH for all of the samples considered for ζ-potential analysis and measures of ζ-potential; sorption experiments in Cd^2+^ aqueous solutions; XPS analyses; and sorption experiments in the presence of Na^+^ and Ca^2+^ (PDF)
