# Supplementary material for: Silver Nanoparticles Supported onto TEMPO-Oxidized Cellulose Nanofibers for Promoting Cd2+ Cation Adsorption
Source: ACS Appl Nano Mater. 2024 Jan 8;7(2):2401–13. doi: 10.1021/acsanm.3c06052 (PMC10825820; doi:10.1021/acsanm.3c06052)
Supplement: Supplementary file 1 — an3c06052_si_001.pdf [file an3c06052_si_001.pdf]

SUPPORTING INFORMATION FOR

"Silver Nanoparticles Supported onto TEMPO-Oxidized Cellulose Nanofibers for Promoting Cd^{2+} Cation Adsorption"

Laura Riva[†], Anna Dotti[†], Giovanna Iucci[‡], Iole Venditti[‡], Carlo Meneghini[‡], Ilaria Corsi[¥], Ivan Khalakhan[§], Gloria Nicastro[†], Carlo Punta[†,*] and Chiara Battocchio[‡,*]

[†] Department of Chemistry, Materials, and Chemical Engineering "G. Natta", Politecnico di Milano and INSTM Local Unit, Via Mancinelli 7, 20131 Milano, Italy

[‡] Department of Science, Roma Tre University, Via della Vasca Navale 79, 00146, Rome, Italy

[¥] Department of Physical, Earth and Environmental Sciences, University of Siena, 53100, Siena

[§] Department of Surface and Plasma Science, Faculty of Mathematics and Physics, Charles University, V Holešovičkách 2, 18000, Prague, Czech Republic

*Corresponding authors: carlo.punta@polimi.it (Carlo Punta) and chiara.battocchio@uniroma3.it (Chiara Battocchio)

Table of Contents

S.1. FTIR-ATR……………………………………………………………………..………………………S-2

Figure S1…………………...…………………………………………..…………………………S-2

S.2. ζ-potential …………….…………………….……………………………...…………S-2

Table S1…………………………………………..……………………...…………S-2

Table S2…………………………………………..……………………...…………S-2

S.3. Sorption experiments in Cd^{2+} aqueous solutions………………………………...…………S-3

Table S3…………………………………………..……………………...…………S-3

S.4. XPS analyses..…………………………………………………..………………………S-4

Table S4..………………………………………………………..………………………S-4

S.5. Sorption experiments in presence of Na^{+} and Ca^{2+}………….……………………...………S-5

Table S5..………………………………………………………..………………………S-5

S.1. FTIR-ATR

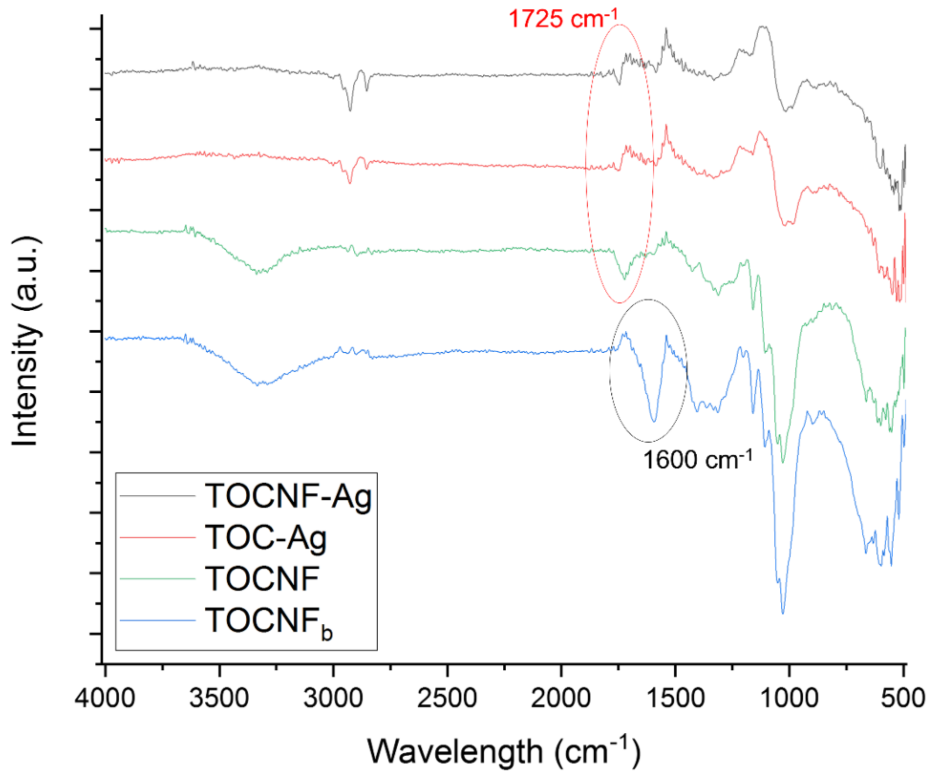

Figure S1. FTIR-ATR spectra of cellulose-based samples.

S.2. ζ-potential

Seven suspensions were prepared by dissolving the cellulose sample in distilled water. Firstly, for each suspension the pH was measured in duplicates at T = 25 °C. The results are reported in **Table S1**.

Table S1. Values of pH for all the sample considered for ζ-potential analysis. The sample TOCNF-Ag represents the material TOCNF-Ag after the interaction with Cd^{2+} solution.

Entry	Sample	pH
1	TOC	4.7
2	TOC_b	6.4
3	TOCNF	4.7
4	$TOCNF_b$	6.5
5	TOC-Ag	6.3
6	TOCNF-Ag	6.9
7	TOCNF-Ag+Cd	6.7

Table S2. Values of ζ-potential for all the cellulose-based materials considered.

Entry	Sample	ζ-potential (mV)
1	TOCn	-26.68 ± 1.64
2	TOCb	-37.18 ± 3.36
3	TOCNFn	-31.59 ± 0.99
4	TOCNFb	-51.73 ± 1.38
5	TOCn-Ag	-43.07 ± 2.17
6	TOCNFn-Ag	-41.57 ± 0.44
7	TOCNFn-Ag+Cd	-25.18 ± 1.00

S.3. Sorption experiments in Cd^{2+} aqueous solutions

Table S3. Values of sorption capacity at equilibrium Q_e (mg g^{-1}) from 150 mg L^{-1} Cd^{2+} aqueous solutions, top rows: TOC, TOC-Ag, TOCNF, TOCNF-Ag; bottom rows: the same four materials pre-treated under alkaline conditions: TOC_b, TOC-Ag_b, $TOCNF_b$ and TOCNF-Ag_b (see text).

Entry	Sample	Qe ± St. Dev. (mg g^{-1})
1	TOC	65.2 ± 2.5
2	TOC-Ag	95.6 ± 1.3
3	TOCNF	78.0 ± 2.0
4	TOCNF-Ag	116.5 ± 4.1
5	TOC_b	101,8 ± 1.3
6	TOC-Ag_b	84.9 ± 3.8
7	$TOCNF_b$	120.6 ± 6.6
8	TOCNF-Ag_b	110.1 ± 6.9

S.4. XPS analyses

Table S4. XPS data analysis results: binding energy (B.E.), full width half maxima (FWHM) and atomic percentage (experimental and theoretical) values for C1s, O1s, Ag3d and Cd3d core level spectra. The proposed assignments are summarized in the last column. The statistical incertitude in semiquantitative evaluation by XPS is estimated as 5% of the calculated value [1].

Sample	Signal	B.E. (eV)	FWHM (eV)	exp. atomic %	th. atomic % (A)	th. atomic % (B)	Assignments (A)
TOCNF	C1s	285.00	1.83	34.4	25	25	C-C (C1)
		286.91	1.83	43.7	41.6	41.6	C-OH, C-O-C (C2)
		288.72	1.83	15.0	> 16.7	16.7	O-C-O, COO^- (C3)
		290.92	1.83	6.9	< 16.7	16.7	COOH (C4)
	O1s	531.40	2.20	30.3	22.2	22.2	C=O
		533.14	2.20	69.7	77.8	77.8	C-O
		535.02	2.20				physisorbed H_2O
TOCNF-Ag	C1s	285.00	1.68	38.7	25	25	C-C (C1)
		286.52	1.68	37.7	41.6	41.6	C-OH, C-O-C (C2)
		288.39	1.68	14.3	> 16.7	16.7	O-C-O, COO^- (C3)
		290.07	1.68	9.4	< 16.7	16.7	COOH (C4)
	O1s	531.80	2.18	31.6	22.2	22.2	C=O
		533.70	2.18	68.4	77.8	77.8	C-O
		535.00	2.18				physisorbed H_2O
	$Ag3d_{5/2}$	368.08	1.54	89.2			Ag(0)
		369.67	1.54	10.8			Ag^{d+}
TOCNF-Ag_Cd	C1s	285.00	1.78	24.4	25	25	C-C (C1)
		286.47	1.78	43.9	41.6	41.6	C-OH, C-O-C (C2)
		288.38	1.78	22.0	> 16.7	16.7	O-C-O, COO^- (C3)
		290.19	1.78	9.7	< 16.7	16.7	COOH (C4)
	O1s	531.04	2.29	33.5	22.2	22.2	C=O
		532.92	2.29	66.5	77.8	77.8	C-O
		535.21	2.29				physisorbed H_2O
	$Ag3d_{5/2}$	368.68	1.74	90.0			Ag(0)
		370.86	1.74	10.0			Ag^{d+}
	$Cd3d_{5/2}$	406.24	2.17	Cd/Ag = 2.0/1.0			Cd^{2+}
TOCNFb	C1s	285.00	1.63	37.2	25	25	C-C (C1)
		286.63	1.63	42.1	41.6	41.6	C-OH, C-O-C (C2)
		288.13	1.63	16.2	> 16.7	16.7	O-C-O, COO^- (C3)
		290.36	1.63	4.5	< 16.7	16.7	COOH (C4)
	O1s	531.03	2.05	21.1	22.2	22.2	C=O
		532.89	2.05	78.9	77.8	77.8	C-O
TOCNFb-Ag	C1s	285.00	1.62	28.1	25	25	C-C (C1)
		286.14	1.62	52.1	41.6	41.6	C-OH, C-O-C (C2)
		288.10	1.62	17.0	> 16.7	16.7	O-C-O, COO^- (C3)
		289.90	1.62	2.9	< 16.7	16.7	COOH (C4)
	O1s	530.81	1.99	15.8	22.2	22.2	C=O
		532.65	1.99	84.2	77.8	77.8	C-O
	$Ag3d_{5/2}$	367.41	1.51	91.4			Ag(0)
		369.39	1.51	8.6			Ag^{d+}

Sample	Signal	B.E. (eV)	FWHM (eV)	exp. atomic %	th. atomic % (A)	th. atomic % (B)	Assignments (A)
TOCNFb-Ag_Cd	C1s	285.00	1.89	40.8	25	25	C-C (C1)
		286.96	1.89	45.0	41.6	41.6	C-OH, C-O-C (C2)
		288.74	1.89	14.2	> 16.7	16.7	O-C-O, COO^- (C3)
				-	< 16.7	16.7	COOH (C4)
	O1s	531.08	2.30	16.8	22.2	22.2	C=O
		533.17	2.30	83.2	77.8	77.8	C-O
	$Ag3d_{5/2}$	368.80	1.72	94.3			Ag(0)
		370.01	1.71	5.7			Ag^{d+}
	$Cd3d_{5/2}$	406.24	2.19	Cd/Ag = 1.4/1.0			Cd^{2+}

[1] Swift, P.; Shuttleworth, D.; Seah, M. P. Practical Surface Analysis by Auger and X-ray Photoelectron Spectroscopy; Briggs, D.; Seah, M. P. Eds.; J. Wiley & Sons: Chichester, 1983 chapter 4.

S.5. Sorption experiments in presence of Na^+ and Ca^{2+}

Table S5. Samples' ICP-OES concentrations for the adsorption experiments conducted in presence of interfering cations (comparison between 24 and 72 h).

	24 h			72 h	
Sample	H_2O_d<br>Qe ± St. Dev. (mg g^{-1})	100 mg L^{-1} Na^+<br>Qe ± St. Dev. (mg g^{-1})	100 mg L^{-1} Ca^{2+}<br>Qe ± St. Dev. (mg g^{-1})	100 mg L^{-1} Na^+<br>Qe ± St. Dev. (mg g^{-1})	100 mg L^{-1} Ca^{2+}<br>Qe ± St. Dev. (mg g^{-1})
TOCNF	78.0 ± 2.0	60.6 ± 0.7	37.0 ± 3.0	49.1 ± 2.5	28.6 ± 0.6
TOCNF-Ag	116.5 ± 4.1	96.8 ± 0.7	62.7 ± 4.5	84.7 ± 3.8	53.5 ± 2.7
$TOCNF_b$	120.6 ± 6.6	98.9 ± 5.2	77.6 ± 13.6	80.3 ± 6.1	49.9 ± 1.6
